# Gene Expression Patterns in Roots of *Camelina sativa* With Enhanced Salinity Tolerance Arising From Inoculation of Soil With Plant Growth Promoting Bacteria Producing 1-Aminocyclopropane-1-Carboxylate Deaminase or Expression the Corresponding *acdS* Gene

**DOI:** 10.3389/fmicb.2018.01297

**Published:** 2018-06-27

**Authors:** Zohreh Heydarian, Margaret Gruber, Bernard R. Glick, Dwayne D. Hegedus

**Affiliations:** ^1^Agriculture and Agri-Food Canada, Saskatoon, SK, Canada; ^2^Department of Biotechnology, School of Agriculture, Shiraz University, Shiraz, Iran; ^3^Department of Biology, University of Waterloo, Waterloo, ON, Canada; ^4^Department of Food and Bioproduct Sciences, University of Saskatchewan, Saskatoon, SK, Canada

**Keywords:** *Camelina sativa*, salinity tolerance, 1-aminocyclopropane-1-carboxylate deaminase, plant growth promoting bacteria, transgenic plants

## Abstract

*Camelina sativa* treated with plant growth-promoting bacteria (PGPB) producing 1-aminocyclopropane-1-carboxylate deaminase (acdS) or transgenic lines expressing *acdS* exhibit increased salinity tolerance. AcdS reduces the level of stress ethylene to below the point where it is inhibitory to plant growth. The study determined that several mechanisms appear to be responsible for the increased salinity tolerance and that the effect of acdS on gene expression patterns in *C. sativa* roots during salt stress is a function of how it is delivered. Growth in soil treated with the PGPB (*Pseudomonas migulae* 8R6) mostly affected ethylene- and abscisic acid-dependent signaling in a positive way, while expression of *acdS* in transgenic lines under the control of the broadly active CaMV *35S* promoter or the root-specific *rolD* promoter affected auxin, jasmonic acid and brassinosteroid signaling and/biosynthesis. The expression of genes involved in minor carbohydrate metabolism were also up-regulated, mainly in roots of lines expressing *acdS*. Expression of *acdS* also affected the expression of genes involved in modulating the level of reactive oxygen species (ROS) to prevent cellular damage, while permitting ROS-dependent signal transduction. Though the root is not a photosynthetic tissue, acdS had a positive effect on the expression of genes involved in photosynthesis.

## Introduction

The ability of *Camelina sativa* (camelina) to grow on marginal lands which are not well-suited for food crops has piqued interest in its development as an industrial oilseed crop for biofuels, bio-lubricants, and animal feed (Blackshaw et al., 2011; Li and Mupondwa, 2014). Moreover, camelina exhibits better agronomic properties, such as enhanced drought and some degree of salinity and cold tolerance, displays early maturation and requires fewer inputs than other oilseed crops, like soybean and canola (Vollmann et al., 1996; Zubr, 2003; Steppuhn et al., 2010). Camelina is also naturally resistant to diseases, such as blackspot (Sharma et al., 2002), blackleg (Li et al., 2005), and stem rot (Eynck et al., 2012), as well as insect pests, such as the flea beetle and diamondback moth (Deng et al., 2002; Henderson et al., 2004; Soroka et al., 2014), that afflict canola.

A genome triplication event was proposed to have given rise to the contemporary *C. sativa* genome (Hutcheon et al., 2010); an assumption supported by genome sequencing (Kagale et al., 2014). Highly undifferentiated polyploidy and little fractionation bias in the *C. sativa* genome presents significant challenges for breeding and genetic manipulation (Kagale et al., 2014; Kanth et al., 2015; Poudel et al., 2015). This situation necessitates exploration of alternate strategies for trait improvement in camelina. One such approach for improving salt tolerance is the application of plant growth-promoting bacteria (PGPB) that are found in association with plant roots (rhizospheric) or within plant tissues (endophytic) (Bacon and Hinton, 2006; Ali et al., 2012), and facilitate plant growth under unfavorable conditions (Glick, 2015). Some PGPB produce 1-aminocyclopropane-1-carboxylate deaminase (acdS). This enzyme converts the ethylene precursor 1-aminocyclopropane-1-carboxylate (ACC) to α-ketobutyrate and ammonia which promotes plant growth, especially during stress conditions thereby reducing the level of stress ethylene to below the point where it is inhibitory to growth (Glick, 1995, 2012; Singh et al., 2015). AcdS has no known function in bacteria; however, its expression in plants or treatment with PGPB strains producing acdS enhances root growth at high salt concentrations in canola, wheat, tomato, barley and red pepper enhances root growth at high salt concentrations (Glick, 1995; Gamalero and Glick, 2015; Olanrewaju et al., 2017; Singh and Jha, 2017; Tavares et al., 2018). In camelina, transgenic lines expressing *acdS* or plants treated with PGPB producing acdS exhibit increased salinity tolerance (Heydarian et al., 2016).

This study examined how gene expression patterns in roots responding to salt stress were affected by the expression of *acdS* under the control of broadly constitutive (CaMV *35S*) or root-specific (*rolD*) promoters in transgenic lines, or by growth in soils treated with PGPB producing acdS.

## Materials and Methods

### Bacterial Strains

Several PGPB that were tested previously for their ability to increase salinity tolerance in camelina (Heydarian et al., 2016) were examined, namely the rhizosphere-associated *Pseudomonas* sp. UW4 (Duan et al., 2013) and two root endophytes, *Pseudomonas migulae* 8R6 and *P. fluorescens* YsS6 (Rashid et al., 2012). Two *acdS* mutant endophytic strains, 8R6M and YsS6M, were also tested (Ali et al., 2014).

### acdS Vector Construction and Plant Transformation

*Camelina sativa* cv. DH55 lines expressing the *acdS* gene from *P.* sp. UW4 under the control of either the double cauliflower mosaic virus (CaMV) *35S* promoter or the *rolD* promoter from *Agrobacterium rhizogenes* were constructed previously (Heydarian et al., 2016).

### PGPB and Salt Treatment

Seeds were sown in soil-less potting mixture (Stringham, 1971). NaCl solutions at 192 and 213 mM were prepared to obtain solutions with electrical conductivities (EC) of 15 dSm^-1^ and EC 20 dSm^-1^ at 20°C, respectively. Bacteria were cultured for 24 h in tryptic soy broth (TSB) containing 100 μg ml^-1^ ampicillin for wild-type strains or 100 μg ml^-1^ ampicillin and 10 μg ml^-1^ tetracycline for the *acdS* mutant strains (Ali et al., 2012). Bacterial cultures were centrifuged at 4,000 ×*g* and resuspended to an OD_600__nm_ = 0.50 ± 0.02 in 0.03 M MgSO_4_. Soil was inoculated with either 2 ml of PGPB or 2 ml of 0.03 M MgSO_4_ (control) at the time of sowing and again 1 week after sowing. 50 ml of tap water (EC 1,248 μSm^-1^) was applied daily to each pot and replaced with 50 ml of saline solution (EC 15 or EC 20 dSm^-1^) 7 and 19 days after sowing for RNA extraction and root measurement, respectively. The accumulation of salt in the pots was controlled by draining and the EC of the drained water was measured weekly. Root material to be measured was harvested 21 days after the initial salt treatment (40-day-old plants) just as plants began to flower at which time the length and dry weights were recorded.

### RNA Sequencing and Data Analysis

Total RNA was extracted from root tissue of three biological replicates for each of the control, *35S::acdS, rolD::acdS* and *P. migulae* 8R6 treatment (12 samples in total) from 28-day-old plants (21 days after salt treatment began and before plants started to bolt) exposed to 15 dSm^-1^ NaCl and from non-stressed plants using the RNeasy Plant Mini Kit (Qiagen Inc.). cDNA libraries were prepared using the TruSeq Stranded mRNA and Total RNA Library Prep kits with TruSeq LT adaptors (Illumina Inc.). The tagged libraries were sequenced using a HiSeq 2500 (Illumina Inc.).

The short-read sequence data from the 12 libraries were deposited in the NCBI GEO database (GSE103720). Trimmomatic (Bolger et al., 2014) was used to discard low-quality reads, trim adaptor sequences, eliminate low quality nucleotides at the beginning or end of the read (PHRED33 quality score of less than 3), and discard short reads (under 21 nt). The retained high-quality reads were mapped to the *C. sativa* reference genome [release 100 (JFZQ00000000.1; Kagale et al., 2014)] and to transcripts available in the NCBI database using STAR RNA-seq aligner (Dobin et al., 2013). Expression levels for each gene were measured as counts (Yaish et al., 2015). For each gene, normalization for library size was performed by dividing the counts by the library size following the method described by Anders and Huber (2010) to yield counts per million (CPM) reads. Further normalization was performed using DESeq to approximate a negative binomial distribution. The differential expression analysis of digital gene expression data software (edgeR) was used to calculate changes in expression between libraries (Robinson and Oshlack, 2010). The Biological Coefficient of Variation (BCV) value was set to 2 according to the software’s instructions. Expression changes were declared to be significant if the multiple test corrected *p*-value and the false discovery rate (FDR) were ≤ 0.05 and the absolute value of log 2 CPM was higher than 0.8. MAPMAN software was used to assign Gene Ontology (GO) terms to unigenes based on molecular function, biological processes and cellular compartment (Klie and Nikoloski, 2012).

### Quantitative Droplet Digital PCR (ddPCR) Analysis

Quantitative ddPCR was performed to compare the expression profiles of select genes as determined by RNA-Seq analysis. Total RNA was extracted from roots of three independent biological replicates (control, *35S::acdS, rolD::acdS* and *P. migulae* 8R6 treatment) using the RNeasy Plant Mini kit (Qiagen) and cDNA synthesized using the SuperScript III First-Strand Synthesis kit (Thermo Fisher Scientific). One ng of cDNA was added to the PCR reaction mixture with 2 X Supermix (Bio-Rad), 10 μM of each primer and 3.25 nM of each probe in a final volume of 20 μl. The sequences of the primers and probes can be found in Supplementary Table S1. Data were analyzed using Quanta-Soft version 1.7.4.0917 (Bio-Rad) and the relative ratio of the candidate gene expression was calculated relative to the expression of an *actin* reference gene by plotting the concentration of FAM over the HEX labeled probe according to the Bio-Rad dd PCR application guide.

### Statistical Analysis

Plant growth measurements were expressed as the mean ± standard error for each treatment. Significant differences between treatments were determined by variance analysis (ANOVA) with a *p*-value of ≤0.05 and pair-wise comparisons were conducted using the Tukey’s Studentized Range (HSD) test using SAS Software 9.3 (TS1M2).

## Results and Discussion

### Root Growth

**FIGURE 1:**
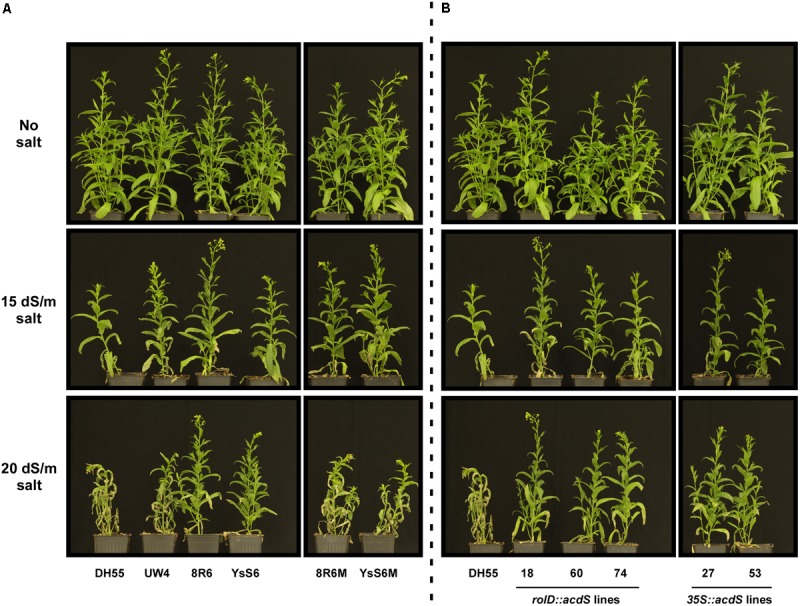
The effect of treatment of soil with PGPB or expression of *acdS* on the growth of *Camelina sativa* in the absence or presence of NaCl (15 and 20 dSm^-1^). **(A)** Soil was treated with buffer (control), *Pseudomonas putida* UW4, *P. migulae* 8R6 or its *acdS-* mutant 8R6M, *P. fluorescens* YsS6 or its *acdS-* mutant YsS6M. **(B)** Lines tested include wild-type *C. sativa* DH55 or independent, single insert, homozygous transgenic lines expressing the *acdS* gene under the control of the root-specific *rolD* promoter or the constitutive CaMV *35S* promoter. Salt was applied 20 days after sowing.

In a previous study, it was demonstrated that camelina lines expressing *acdS* or treated with PGPB were more tolerant to a salt mixture that simulated naturally saline soils found on the Canadian/North American prairies (Heydarian et al., 2016). The current study used NaCl at the same EC values as this provided for better comparision to most other published studies on salinity tolerance which use only NaCl. As per our previous study with the simulated saline solution, inoculation of soil with PGPB increased salt (NaCl) tolerance in *C. sativa*, in particular with the endophytic 8R6 and YsS6 strains. This was not observed with bacteria in which the *acdS* gene had been disrupted and was most noticeable at the higher salt concentration (20 dSm^-1^) (**Figure 1**). Expression of the *acdS* gene under the direction of either the *rolD* or *35S* promoters in transgenic lines improved salt tolerance at both salt concentrations tested.

**FIGURE 2:**
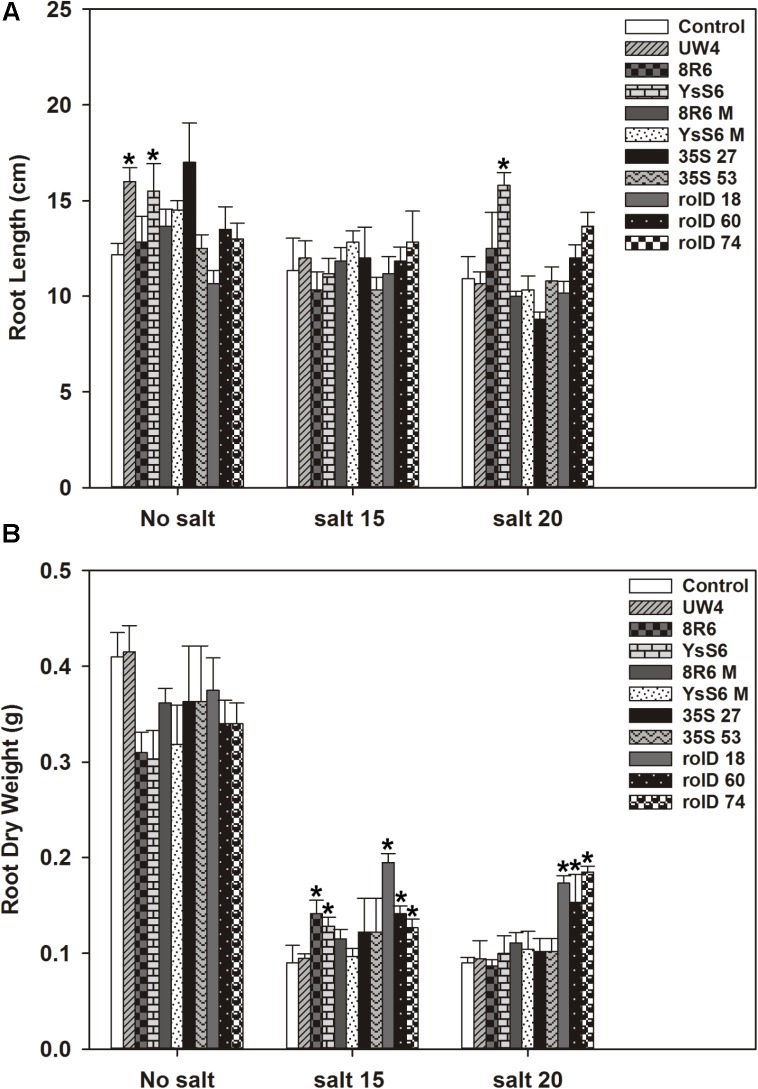
The effect of *acdS* expression or growth in soil inoculated with *P. migulae* 8R6 on root length **(A)** and dry weight **(B)** of *C. sativa* in presence of salt (NaCl; 15 and 20 dSm^-1^). Soil was treated with buffer (control), *P*. sp. UW4, *P. migulae* 8R6 or its *acdS* mutant 8R6M, *P. fluorescens* YsS6 or its *acdS* mutant YsS6M. Transgenic lines tested were single insert, homozygous lines expressing the *acdS* gene under the control of the root-specific *rolD* promoter or the constitutive CaMV *35S* promoter. Salt was applied 19 days after sowing and root material harvested 20 days afterward. Error bars indicate standard error (*n* = 10). A two- way ANOVA and Tukey post-test were used to detect significant differences between groups. Asterisks (^∗^) above bars indicate values that were significantly different (*p* < 0.05) from the control.

In the absence of salt, root length was not significantly affected in the *acdS* transgenic lines; however, the application of PGPB (*P*. sp. UW4 or *P. fluorescens*YsS6) to the soil increased root length significantly (**Figure 2**). Root dry weight was not affected by *acdS* expression or PGPB treatment in the absence of salt. Root dry weight decreased as salt (NaCl) concentration increased; however, the decline in root weight was significantly less in plants expressing *acdS* under the *rolD* promoter at the 15 and 20 dSm^-1^ salt levels. Root weight was also less severely affected in plants treated with the endophytic PGPB strains (8R6 and YsS6) at 15 dSm^-1^ salt, but not so with the corresponding *acdS-* mutants.

Root biomass is generally less affected by excess salinity than above-ground structures (Munns and Tester, 2008). Rewald et al. (2011) found that while the fine root biomass of both salt-sensitive and salt-resistant olive varieties was significantly reduced under saline conditions, the decrease in root biomass was greater in the salt-sensitive variety. In camelina, lines expressing *acdS*, in particular those using the *rolD* promoter, exhibited less decline in root length and weight under conditions that mimicked natural saline soil (Heydarian et al., 2016).

### Global Changes in Camelina Transcription in Response to Salt Treatment

To further explore the effect of acdS on the response of camelina to salinity stress, the root transcriptome of the wild-type DH55 line was compared to lines expressing *acdS* under the control of the constitutive CaMV *35S* or the root-specific *rolD* promoters when grown under saline conditions. In roots, the expression of the *acdS* gene as based on RNA-Seq data was 40 times higher under the control of the CaMV *35S* promoter than under the *rolD* promoter. Roots from plants treated with *P. migulae* 8R6 were also examined under the same conditions. Both of the endophytic PGPB strains (8R6 and YsS6) improved salt tolerance and reduced the impact of salt on root growth. 8R6 was selected for this experiment as it had a slightly greater protective effect on shoot and root growth under simulated natural saline conditions as determined previously (Heydarian et al., 2016).

**FIGURE 3:**
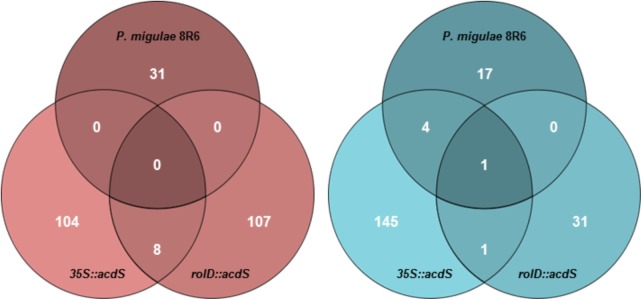
Venn diagrams depicting the number of genes that were differentially expressed in roots of *C. sativa* lines expressing *acdS* under the control of the root-specific *rolD* promoter or the constitutive CaMV *35S* promoter or treated with *P. migulae* 8R6 in the presence of salt. Up-regulated **(left-hand panel)** and down-regulated **(right-hand panel)** genes in roots 28-day-old plants treated with salt (NaCl; 15 dSm^-1^) for 3 weeks. Genes with an FDR and *P*-value ≤ 0.05 and an absolute value of log 2 FC higher than 0.8 were considered significant.

In total, 31 genes were up-regulated and 22 genes were down-regulated in roots from plants treated with *P. migulae* 8R6, while 112 genes were up-regulated and 147 genes were down-regulated in the *35S::acdS* line, and 114 genes were up-regulated and 32 genes were down-regulated in the *rolD::acdS* line (FDR ≤ 0.05 and expression >1.7-fold; Supplementary Table S2) in the presence of salt compared to the wild-type line cv. DH55 (**Figure 3**). Of these, 18 genes were assigned to *C. sativa* Genome I, 19 genes to Genome II, and 16 genes to Genome III in the *P. migulae* 8R6-treated plants. In the *35S::acdS* line, 82 genes were assigned to Genome I, 79 genes to Genome II and 94 genes to Genome III, while in the *rolD::acdS* line, 48 genes were assigned to Genome I, 37 genes to Genome II and 60 genes to Genome III. In the *35S::acdS* line, approximately 15% more genes were differentially regulated on Genome III in response to salinity stress than on the other two genomes. However, in the *rolD::acdS* line, the number of differentially regulated genes was about 20% greater on Genome I and 40% greater on Genome III compared to Genome II in response to salinity stress. Further evidence for partial genome partitioning was found by examining the expression of homeologous genes. In roots of plants treated with *P. migulae* 8R6, 6 differentially expressed homeologous genes (15%) were assigned to 2 genomes, while only 3 homeologous genes (7.5%) were assigned to all three genomes with the remainder being assigned to only 1 genome (ca. 80%). In the *35S::acdS* line, 46 genes (23%) were assigned to two genomes and 21 genes (10%) to all three genomes, while in the *rolD::acdS* line, 26 genes (21%) were assigned to 2 genomes and 21 genes (17%) were assigned to all 3 genomes.

Interestingly, in roots of plants over-expressing *acdS* under the CaMV *35S* promoter, Csa07G019240 from Genome II was down-regulated, while its homeolog Csa05G053740 from Genome III was up-regulated. These genes are orthologous to *Arabidopsis thaliana* AT1G58200 which encodes an MSCS-like 3 transmembrane transporter involved in ion transport and plastid organization. Changes in the expression of AT1G58200 were observed in response to osmotic stress and seedlings of an MSCS-like 3 mutant (*ms-l 3*) exhibited several hallmarks of drought or salinity stress under non-stressful conditions (Wilson et al., 2014). The only gene that was commonly regulated by all treatments was Csa09g083890 (protein with an unknown function) which was down-regulated 14- (*rolD::acdS*) to 100-fold (*35S::acdS* and *P. migulae* 8R6-treated). To validate the RNA-Seq results, a select group of differentially expressed genes (both up- and down-regulated) were selected for quantititive dd PCR analysis (Supplementary Figure S1). The expression profiles of the genes as examined by both methods were highly similar. A complete annotation of the genes that were differentially expressed in roots of these lines can be found in Supplementary Table S2 and are discussed in more detail below. Gene expression heat maps superimposed on MAPMAN GO pathways are provided Supplementary Figure S2.

### Functional Classification of Systems and Biochemical Pathways Affected by Salt Treatment

The key to salt tolerance in plants is to successfully balance osmotic adjustment, ion requirements, the energy pool and the amount of Na^+^ entering or effluxing from root cells in order to maintain a low Na^+^:K^+^ ratio in the cytosol (Redwan et al., 2016). To achieve this, many different processes need to be integrated to remove or compartmentalize excesss ions, to synthesize organic osmolytes to maintain osmotic balance, and to provide sufficient energy for plant growth and seed production. Below, the response of camelina roots to salinity stress and how this may have been altered/enhanced by expression of *acdS* or treatment with PGPB as inferred from gene expression data is examined.

#### Carbon and Energy Metabolism

##### Photosynthesis

Though not a photosynthetic tissue, genes encoding proteins involved in aspects of photosynthesis are expressed in roots and are repressed under stress, which in the case of phosphate deficiency leads to sustained root growth (Kang et al., 2014). In the current study, Csa14g024950 (chlorophyllase 1) was down-regulated 16-fold after salt treatment in roots of plants treated with *P. migulae* 8R6 compared to the control. This enzyme is involved in chlorophyll degradation and expression of the corresponding gene is induced rapidly by methyl-jasmonate, a known promoter of senescence and chlorophyll degradation (Zhou et al., 2008; TAIR database). During salt stress, genes related to photosystems I and II were up-regulated in roots of plants expressing *acdS* compared to the control. For example, Csa04G046670 and Csa05G020850 (homeologs corresponding to *A. thaliana* AT2G34420 in photosystem II) and Csa06G029390 (corresponding to *A. thaliana* AT3G54890 in photosystem I) were up-regulated threefold in *35S::acds* roots, while Csa01g026130 (chlorophyll A–B binding family protein) was up-regulated sixfold in *rolD::acdS* roots. In other studies, palm (*Phoenix dactylifera* L.) exposed to salinity stress also exhibited changes in the expression of genes involved in photosynthesis in roots (Yaish et al., 2017). Why these genes are expressed in roots at all, why their expression is altered in response to stress and how this impacts salinity tolerance requires further investigation.

##### Carbohydrate metabolism

Three of the six genes that were commonly up-regulated in *35S::acdS* and *rolD::acdS* roots (Csa16G005800, Csa06G020990, and Csa05G091890) (Supplementary Table S2) are involved in balancing cellular sugar levels (Hanson et al., 2008; Wohlbach et al., 2008). Another encodes an enzyme involved in pantothenate (vitamin B5) synthesis (Csa04g065150), the key precursor for the biosynthesis of coenzyme A (CoA), which is important for the synthesis and metabolism of proteins, carbohydrates, and fats (Costaglioli et al., 2005; Leonardi and Jackowski, 2007).

In general, the expression of genes involved in major carbohydrate metabolism/synthesis was reduced as a consequence of salinity stress. However, the expression of the genes involved in minor carbohydrate metabolism, such as raffinose biosynthesis (an osmoprotectant and signaling molecule to increase salinity tolerance), were up-regulated in roots of plants expressing *acdS* during salt stress. In *35S::acdS* roots, 2 genes encoding sucrose synthase 5 (Csa10G040720 and Csa11G050910) were down-regulated two–fourfold, respectively, while three genes encoding sucrose-proton symporter 1 (Csa16G034520, Csa09G075220, and Csa07G038820) and 1 gene encoding beta-glucosidase 2D (Csa15G017920) were up-regulated two–threefold. Csa13G023210 and Csa08G014090 (raffinose synthase) were up-regulated twofold and Csa09G018520 (galacturonosyl transferase-like 10) was up-regulated 3.5-fold. In *rolD::acdS* roots, a gene encoding beta-amylase (Csa12g049650) was up-regulated twofold and a gene encoding beta-glucosidase 18 (Csa17g092400) was up-regulated fivefold during salt stress. In *A. thaliana*, this enzyme (AtBG1) hydrolyzes glucose conjugated to abscisic acid (ABA) to produce active ABA. In creeping bentgrass, over-expression of *AtBG1* increases drought resistance (Han et al., 2012), while several biotic and abiotic stress induce its expression in *A. thaliana* (TAIR database).

In roots of *35S::acdS* plants, Csa04G035130 (aldolase-type TIM barrel family protein) was up-regulated 19-fold. This enzyme converts glyceraldehyde-3-phosphate into fructose-1,6-biphosphate which can then used to form glucose-6-phosphate for the production of minor carbohydrates, such as raffinose and phosphoinositol. In roots of plants treated with *P. migulae* 8R6, Csa20g015440 (myo-inositol-1-phosphate synthase 3) was up-regulated threefold, while Csa09g086550 and Csa07g052330 (myo-inositol-1-phosphate synthase) was up-regulated fivefold in *rolD::acdS* plants.

In *35S::acdS* plants, some genes involved in galactose metabolism (Csa19G003320 and Csa15G001220) and galactinol (Csa07G061680) synthesis were down-regulated. Drought, salinity and cold stress induce the expression of genes encoding galactinol synthase which plays a key role in the accumulation of galactinol and raffinose under abiotic stress conditions. Over-expression of a gene encoding this enzyme, *AtGolS2*, improved drought tolerance in *A. thaliana* (Taji et al., 2002), while over-expression of a gene encoding myo-inositol-1-phosphate synthase improved salt and drought tolerance in sweet potato (Zhai et al., 2015). The seemingly contradictory expression of genes involved in minor carbohydrate metabolism in the *35S::acdS* line may be one the reasons why lines expressing *acdS* under the control of the root-specifc *rolD* promoter exhibit better tolerance to salinity (Heydarian et al., 2016).

#### Cell Wall Biosynthesis/Turnover

Sustained cellulose synthesis, cellulose deposition and cell wall biogenesis are important features of salt tolerance (Zhang et al., 2015). In both the *rolD::acdS* and *35S::acdS* lines, Csa10G019050 (cellulose synthase-like G3) was up-regulated in roots in response to salt stress compared to the wild-type line. In addition, Csa10G003280 (xyloglucan endotransglucosylase/hydrolase 7 involved in cell wall biogenesis and organization) was up-regulated fourfold in the *35S::acdS* line. In the *rolD::acdS* line, Csa13g008910 and Csa20g009230 (polygalacturonase inhibiting protein 2; PGIP2) were up-regulated three and sixfold, respectively. PGIPs inhibit the activity of pectin-degrading enzymes, such as those involved in cell wall turnover or those produced by plant pathogens. An *A. thaliana* line with a mutation in a gene encoding PGIP (AT5G06870) exhibited severe damage to the root tip in a low calcium and low pH medium (TAIR database). Interestingly, genes involved in cell enlargement were mostly down-regulated in the *35S::acdS* line in response to salt stress. For example, Csa03G027580 (polygalacturonase 3), Csa09G011170 (polygalacturonase 1), Csa20G032840 (invertase/pectin methylesterase inhibitor) were down-regulated 2- to 2.5-fold. Orthologs of these genes are involved in cell enlargement and maintenance of cell wall integrity (Xiao et al., 2014; Park et al., 2015). *A. thaliana* mutants lacking PGX1 (polygalacturonase 1) or invertase/pectin methylesterase inhibitor have reduced hypocotyl elongation (Xiao et al., 2014), while lines over-expressing these genes display increased root length (Lionetti et al., 2007). None of the genes involved in pectin metabolism were differentially expressed in response to salt stress in the *rolD::acdS* line or in roots of plants treated with *P. migulae* 8R6. These results are in agreement with the physiological experiments which showed that *35S::acdS* lines exhibited a slight decrease in root length or dry weight during salt treatments, while *rolD::acdS* lines and lines treated with PGPB exhibited increased root length or root dry weight compared to the control (**Figure 2**).

#### Phytohormones

Hormones are fundamental to the plant’s ability to adapt to environmental changes. While acdS directly impacts ethylene production, the expression of *acdS* was also found to alter the expression of genes involved in other hormone pathways as described below.

##### Auxin

The concentration of auxin (IAA) in plants is regulated by biosynthesis, transport and activation (Tanaka et al., 2014). It has been suggested that tryptophan released by roots is used by PGPB to synthesize IAA, some of which is then taken up by the plant to promote root growth (Gamalero and Glick, 2015). However, in the current study plant genes involved in tryptophan synthesis or IAA production were not differentially expressed during salt treatment in plants treated with *P. migulae* 8R6.

Ethylene inhibits root growth, root elongation and root hair formation and elongation through the accumulation of auxin in the primary root tip (Swarup et al., 2002; Alonso et al., 2003; Stepanova et al., 2005). Several genes involved in auxin metabolism were differentially expressed in the *35S::acdS* line, these included Csa14G007040 and Csa17G009070 (uridine diphosphate glycosyltransferase 74E2 involved in auxin production), and 6 genes related to auxin inactivation through conjugation (UDP-glucosyl transferase 74D1, 73C7) which were up-regulated threefold during salt treatment. Uridine diphosphate glycosyltransferase 74E2 acts on indole-3-butyric acid (IBA) and affects auxin homeostasis. Transcript and protein levels of this enzyme are strongly induced by H_2_O_2_ which may allow integration of reactive oxygen species (ROS) and auxin signaling. *A. thaliana* lines over-expressing *UGT74E2* exhibited improved survival during drought and salt stress (Tognetti et al., 2010). Csa13G050980 encodes an auxin-induced protein involved in lateral root morphogenesis and was the only auxin-related gene that was down-regulated in the *35S::acdS* line. A gene encoding an auxin-responsive GH3 family protein (Csa17g080380) was the only auxin-related gene to be differentially expressed in both transgenic *acdS* lines. Five other camelina genes encoding auxin-responsive GH3 family proteins were up-regulated 10- to 21-fold only in the *35S::acdS* line.

##### Abscisic acid (ABA)

The relationship between ethylene and ABA on root growth, especially under salinity response, is not very clear. In rice, ABA enhances inhibition of root growth by ethylene (Tao et al., 2015), whereas in other plants (*A. thaliana*, maize, barley and tomato) ABA accumulation promotes shoot and root growth through a negative interaction with ethylene. In the latter species, ABA prevents excess ethylene production that would otherwise inhibit root elongation during stress (Sharp and LeNoble, 2002).

In the current study, several genes involved in ABA synthesis and signaling were differentially expressed during salt treatment in the roots of plants treated with *P. migulae* 8R6. These included a gene encoding 9-*cis*-epoxycarotenoid dioxygenase 3 (Csa19g021150), the rate-limiting enzyme in ABA biosynthesis, which was down-regulated 2.5-fold. In contrast, Csa05g012440, which encodes the PYL6/RCAR regulatory component of ABA receptor 9, was up-regulated fourfold. PYR/PYL/RCAR family proteins function as ABA sensors and mediate signal transduction through ABA-dependent regulation of ABI1 and ABI2. In *A. thaliana*, PYL6 induces stomatal closure, but does not inhibit seed germination or root growth (Takeuchi et al., 2015). In roots of salt-treated *rolD::acdS* plants, Csa17g016450, Csa14g015170, and Csa03g015980 (glycosyl hydrolase family 32) were up-regulated two–threefold. This enzyme is involved in ABA-activated signaling, primary root development and root biomass accumulation (Chen et al., 2016; Leskow et al., 2016). It should be noted that ABA synthesized in the roots is unlikely to remain there as it transported upward through the xylem to the leaves where it induces stomatal closure under stress conditions.

##### Plant defense hormones

###### Ethylene

Ethylene signaling modulates the response to salt stress at several levels, including the cell membrane (receptors), cytoplasm (signaling) and nucleus (transcription) (Cao et al., 2008).

In roots of plants treated with *P. migulae* 8R6, several genes involved in ethylene signal transduction were differentially expressed in response to salt treatment, for example, five genes encoding the ethylene response factors ERF6, ERF105, and ERF106, were up-regulated three–fourfold (Supplementary Table S2). ERFs regulate the response to pathogen attack by binding to a *cis*-acting promoter element, the GCC box; however, they can also bind to dehydration-responsive elements and act as a regulatory hub in the response to hormones, biotic and abiotic stresses, including salinity stress (Müller and Munné-Bosch, 2015). ERF6 has been implicated in stress tolerance and growth inhibition in adapting leaf growth to environmental changes; activation of the stress tolerance genes by ERF6 occurs independently of ERF6-mediated growth inhibition (Dubois et al., 2013). ERF105 is a cold-regulated transcription factor linked to the C-repeat binding factor (CBF) regulon mediating response to cold (Bolt et al., 2017) and ectopic expression increases tolerance to salt stress (Chinnusamy et al., 2010). PGPB can effectively protect against different stresses, including flooding, high salt, drought and low temperature (Glick, 2014, 2015). Induction of genes, like *ERF105*, in plants treated with PGPB may explain why these bacteria induce tolerance in plants subjected to a wide variety of stresses. Another gene that was up-regulated in roots of plants treated with *P. migulae* 8R6 was Csa20g007990 which encodes anthranilate synthase 1 (ASA1), an enzyme involved in cross-talk between the ethylene and auxin pathways. *ASA1* expression is induced by ethylene, downstream of ERF1, and *A. thaliana* lines over-expressing *ERF1* were more tolerant to drought and salt stress (Cheng et al., 2013).

In roots of the *35S::acdS* line subjected to salt treatment, Csa04G062700 (ERF13) was up-regulated. The only role recognized for ERF13 is in transferring the wound signal from roots to shoots (Sogabe et al., 2011). In roots of the *rolD::acdS* line, genes encoding other ERFs were up-regulated, for example 2 genes (Csa17g069360 and Csa03g046520) encoding ERFB-4 or RAP2.6 were up-regulated around eightfold. In *A. thaliana*, RAP2.6 participates in the response to salt and osmotic stress through the ABA-dependent pathway and expression of *RAP2.6* was highly induced in seedlings under salt stress (Zhu et al., 2010).

###### Jasmonic acid (JA)

Jasmonic acid-mediated adaptation to salinity stress occurs in barley and sweet potato (Walia et al., 2007; Zhang et al., 2017); however, none of the genes involved in JA biosynthesis or signaling were differentially expressed during salt treatment in roots of the *35S::acdS* line or in plants treated with *P. migulae* 8R6. Conversely, several genes involved in JA signaling were differentially expressed in the *rolD::acdS* line, including Csa05g020670 [jasmonate-ZIM-domain protein (JAZ) 7], Csa16g032280 (JAZ 9), and Csa20g01859, Csa20g018580, and Csa08g005100 (JAZ 10) which were up-regulated between two and fourfold. JAZ proteins are key negative regulators of jasmonate signaling (Oh et al., 2013). A gene encoding allene oxide (12-OPDA) synthase (Csa20g066350) was up-regulated twofold. 12-OPDA is a precursor of JA and dehydration stress uncouples the conversion of 12-OPDA to JA. Plants producing higher levels of 12-OPDA exhibit enhanced drought tolerance and reduced stomatal aperture size (Savchenko et al., 2014). Other genes important in JA biosynthesis that were up-regulated in salt-treated *rol::acdS* roots included Csa08g057450, Csa20g009360 and Csa13g009030 (sulfotransferase 2A), Csa11g035060 (hydroperoxide lyase 1) and Csa06g030650 (jasmonate-regulated gene 21).

Sulfotransferase 2A and hydroperoxide lyase 1 inactivate and, therefore, regulate the level and biological activity of JA (Gidda et al., 2003; Nilsson et al., 2016).

The relationship between JA and the response of roots to biotic/abiotic interactions is complicated. JA perception is required for the induction of systemic resistance by root-associated *P. fluorescens* (Pieterse et al., 1998). JA signaling also mediates aluminum exclusion and causes root growth inhibition under aluminum stress (Yang et al., 2017). However, in the current study, negative regulators of JA signaling (*JAZ* genes) were up-regulated by salt stress, which is also the situation in *A. thaliana* roots and in lines with mutations in JA-related genes (*AOS, COI1, JAZ3*, and *MYC2/3/4*). Cortical cells in the root elongation zone are also significantly longer in these mutants compared with wild-type plants under salt stress (Valenzuela et al., 2016). It appears that very tight regulation of JA signaling is occurring in the *rolD::acdS* plants as genes involved in both JA synthesis and negative regulation of its effects (JAZ proteins) were induced. This may be why the *rolD::acdS* plants are the more tolerant to salt stress than the *35S::acdS* lines or plants treated with PGPB (Heydarian et al., 2016).

In general, based on gene expression data, the acdS-producing *P. migulae* 8R6 strain positively affected ethylene signaling by increasing the expression of genes involved in ethylene-activated signaling pathways, namely those encoding ERFs. In addition, ABA production and signaling was also positively affected by *P. migulae* 8R6 treatment during salt stress. In transgenic lines expressing *acdS*, the effect was dependent on the promoter. In plants where *acdS* was under the control of the root-specific *rolD* promoter, which produces 40 times less *acdS* transcript than lines using the CaMV *35S* promoter, expression of the genes involved in JA signaling and JA biosynthesis were the most affected. However, in plants in which *acdS* was under the control of the *35S* promoter the expression of the genes involved in auxin inactivation or which negatively affect auxin signaling were affected.

#### Secondary Metabolism

In roots of plants grown in soil treated with *P. migulae* 8R6, a gene encoding ribonuclease 1 (Csa06g002980) was down-regulated 16-fold. This enzyme inhibits production of anthocyanin and is important for nutrient recycling as expression of the corresponding gene is responsive to both phosphate and phosphite starvation (LeBrasseur et al., 2002; TAIR database). In roots of *35S::acdS* and *rolD::acdS* plants treated with salt, genes related to the production of glucosinolates, anthocyanins, flavonoids and/or isoflavonols were up-regulated three–ninefold. Only one gene related to secondary metabolism was down-regulated in the *acdS* lines, this being Csa09g009000 (terpene synthase-like) in roots of the *rolD::acdS* line. This enzyme was shown to be involved in root development in *A. thaliana* as over-expression of the corresponding gene impairs root elongation (Chen et al., 2004).

#### Stress Response

##### Chaperones and heat shock proteins

Three camelina genes encoding chaperones, Csa11g024410 (HSP20) and Csa07g039500 and Csa16g035090 (HSP40), were up-regulated (five–sixfold) in roots of plants treated with *P. migulae* 8R6. A gene encoding an HSP20-like chaperone (Csa02G043250) was up-regulated in the *35S::acdS* line, while a gene encoding a metallo-chaperone (Csa03G026830), which is involved in the transport of metallic ions inside the cell, was down-regulated. No genes encoding chaperones or heat shock proteins were differently regulated in roots of the *rolD::acdS* line during salt treatment.

##### Redox state

H_2_O_2_ and superoxide anion are the main ROS affecting root growth; however, H_2_O_2_ also regulates cell extensibility as it is involved in cross-linking different cell wall-associated molecules. In contrast, excessive levels of H_2_O_2_ inhibit root elongation (Liszkay et al., 2004; Dunand et al., 2007). ROS signaling also regulates the expression of salt tolerance genes, a process that may be amplified by NADPH oxidases (Mittler et al., 2004). Scavenging of excess ROS is necessary to prevent cellular damage and, therefore, systems are in place to finely control ROS levels (Pang and Wang, 2008).

Several genes related to redox state were differentially regulated in both the transgenic lines expressing *acdS* lines and in plants treated *P. migulae* 8R6. In roots of PGPB-treated plants, Csa15g001040 and Csa01g001030, which encode alpha-dioxygenase 1 involved in protection against oxidative stress and cell death, were up-regulated ninefold. Plant fatty acid α-dioxygenases (α-DOX) are oxylipin-forming enzymes that participate in developmental processes while also providing protection from oxidative stress induced by biotic and abiotic stresses (Ponce de León et al., 2002). Studies in *A. thaliana* demonstrated that α*-DOX1* is controlled by ABA and SA, and α*-dox1* mutants were more sensitive to salinity (Ponce de León et al., 2002; Aung, 2009).

In roots of *35S::acdS* plants treated with salt, many genes related to the redox state were down-regulated. These included 5 genes encoding peroxidases, a gene encoding thioredoxin H-type 7 (Csa16G017640), and 5 genes encoding cytochrome p450 enzymes involved in oxidation-reduction processes. In contrast, the expression of Csa06G023070 (peroxidase PRX34) and 5 other genes encoding cytochrome p450 enzymes were up-regulated. Four genes encoding cytochrome p450 enzymes and a gene encoding peroxidase CB (Csa04g034500) were up-regulated in roots of the *rolD::acdS* line. Interestingly, the expression of Csa03g055440 (peroxidase superfamily protein) was down-regulated in roots of *rolD::acdS* plants, whereas the expression of its homeolog (Csa17G083270) was down-regulated in the roots of the *35S::acdS* line. This indicates that the expression of homeologous genes and/or paralogous genes with the same function is dependent upon the level of acdS, and presumably the level of ethylene, in response to salt stress. In general, it appears that acdS modulates the level of ROS in roots undergoing salt stress to enhance ROS signaling, but also alters the expression of genes encoding enzymes/proteins involved in preventing ROS damage to cells.

##### Biotic stress

The deployment of defenses against biotic and abiotic stress overlaps; however, activation of defenses specific to the stress being encountered is crucial to maximize plant fitness while not unnecessarily compromising growth and development (Huot et al., 2014). In this regard, most genes related to the defense response to biotic stress were down-regulated in roots of the *35S::acdS* line during salt treatment; these included Csa12G010010 and Csa10G008480 (pathogenesis-related 1 protein PR1), Csa05G006780 (chitinase), and Csa13G048800 (osmotin 34). In contrast, Csa10G040380 (defensin 1.2) and Csa06G020990 (glutamine-dependent asparagine synthase CaAS1) were up-regulated. CaAS1 is required for asparagine synthesis and disease resistance in *A. thaliana* (Hwang et al., 2011), while over-expression of the gene encoding defensin 1.2 leads to a reduction in symptoms caused by the non-host pathogen *Cercospora beticola* (De Coninck et al., 2010). Contrary to the *35S::acdS* line, most biotic stress-related genes were up-regulated in the *rolD::acdS* line under salt stress, including Csa07g037710 (MLP-like protein 28), Csa05g006860 (scorpion toxin-like knottin), and Csa16g032520 (MLP-Like Protein 34). Another homeolog encoding defensin 1.2 (Csa07g047380) was also up-regulated in the *rolD::acdS* line. The only biotic defense gene that was down-regulated in the *rolD::acdS* line was Csa17g055720, which encodes a wound-responsive protein. In roots of plants treated with *P. migulae* 8R6, genes encoding basic secretory protein (Csa15g071190) and phytoalexin-deficient 3 (Csa10g045180) were up-regulated, but a gene encoding a legume lectin family protein involved in the SA-related defense response was down-regulated. *Phytoalexin-deficient 3* is highly expressed in response to pathogens and specific abiotic triggers (Chapman et al., 2016). Interestingly, genes encoding thionins were down-regulated in both the PGPB-treated plants and the *35S::acdS* line, but were up-regulated in the *rolD::acdS* line. Thionins are small, sulfur-rich proteins that have been suggested to be involved in defense against bacterial and fungal pathogens (Bohlmann, 1994; Nawrot et al., 2014).

Down-regulation of genes involved in biotic stress was expected since ethylene is a major player in defense signaling and functions as an important modulator of plant immunity during host–pathogen interactions (Pieterse et al., 2012). However, it seems that in the *rolD::acdS* line, which expresses markedly lower levels of *acdS* (Heydarian et al., 2016), the amount of ethylene remaining may better balance the expression of genes involved in abiotic and biotic stress responses so as to not compromise growth or negatively impact the plant’s defense systems.

##### Abiotic stress

With the exception of genes encoding heat shock proteins or proteins involved in redox state, no other genes related to a specific abiotic stress were differentially regulated in response to salt in roots of the *35S::acdS* line or in plants treated with *P. migulae* 8R6. Conversely, in the *rolD::acdS* line, a gene encoding annexin 4 (Csa04g052610) orthologous to the *A. thaliana* AT2G38750 gene induced in response to ABA, cold, heat, osmotic, and salt stress, as well as Csa13g018780 (cold-regulated protein COR6.6) were up-regulated twofold.

#### Signaling

##### Transcription factors (TFs)

Adaptation to salinity stress involves several different physiological mechanisms whose deployment is regulated and coordinated at the transcriptional level by specific TFs. In plants treated with *P. migulae* 8R6, only a single TF gene, that encoding the zinc finger TF ZAT10 (Csa14g035300), was up-regulated. In *A. thaliana*, ZAT10/12 mediates the anti-oxidant defense and maintains ion homeostasis during salt stress (Xie et al., 2012).

A few genes encoding TFs, such as Csa19G001330 (NAC44), Csa10G003940 (bHLH orthologous to AT4G36930) and Csa04G046570 (phytochrome interacting factor 3-like 5 orthologous to AT3G59060) were up-regulated in roots of the *35S::acdS* line in response to salt. The TF encoded by AT3G59060 is involved in ethylene and auxin signaling, as well as red light photo-transduction (Miyazaki et al., 2016). The TF encoded by AT4G36930 is a negative regulator of seed germination and is involved in regulation of the circadian rhythm, as well as carpel, flower and fruit development in *A. thaliana* (Makkena and Lamb, 2013). About 15 genes encoding TFs belonging to the WRKY, zinc finger, basic helix-loop-helix (bHLH), MYB and homeobox families were down-regulated in roots of the *35S::acdS* line. Among them were those encoding WRKY51 (Csa02G073920 and Csa18G038500) and WRKY54 (Csa05G011960) that were down-regulated 2- to 11-fold. WRKY51 is involved in the JA-related defense response pathway, while WRKY54 is a negative regulator of leaf senescence involved in osmotic stress tolerance (Li et al., 2013).

In roots of the *rolD::acdS* line, Csa01g002130, Csa15g002200, and Csa19g004650 (Lateral Organ Boundaries domain-containing 41) were down-regulated, while other TF genes were up-regulated, for example Csa05g064600 (MYB50; 14-fold) and Csa11g025000 (MYB-like 102; 6-fold). Members of the Lateral Organ Boundaries family are found in the base of lateral roots and are important in lateral organ development, in particular for a subset of JA-regulated mediated defenses (Shuai et al., 2002; Thatcher et al., 2012). MYB50 is involved in cell differentiation, regulation of stomatal movement, and response to auxin, gibberellin, JA and SA. AtMYB102 is expressed in response to both wounding and osmotic stress (Denekamp and Smeekens, 2003). Genes encoding MYB2 (Csa04g066350) and MYB29 (Csa08g056690) were up-regulated threefold in the *rolD::acdS* line. MYB2 is involved in regulating salt- and dehydration-responsive genes and MYB29 acts as a negative regulator of mitochondrial stress and is involved in glucosinolate biosynthesis (Yu et al., 2012; Araki et al., 2013). Nine genes encoding bHLH TFs and five genes encoding APETALA2/ethylene response factor (AP2/ERF) proteins, including Csa17g069360 and Csa03g046520 encoding Ap2.6, were up- regulated two–ninefold in the *rolD::acdS* line. AP2/ERF TFs are mediators of stress responses and developmental programs. *AP2.6* is responsive to salt and drought stress, possibly through an ABA-dependent pathway (Zhu et al., 2010).

#### Transporters Involved in Ion Homeostasis

Many quantitative trait loci (QTL) associated with salt-tolerance in plants map to genes involved in ion transport (Kader and Lindberg, 2010). Influx of Na^+^ causes membrane depolarization and a significant outward K^+^ current (Anschütz et al., 2014; Véry et al., 2014). About 10 genes related to ion transport were down-regulated and 14 genes up-regulated in *35S::acdS* plants in response to salt stress compared to the control. However, while a few genes encoding transporters were differentially expressed in the *rolD::acdS* line or plants treated with *P. migulae* 8R6, none were related to ion transport. This suggests that the level of acdS, and therefore ethylene, influences ion transport dynamics in root cells.

K^+^ uptake generally occurs via active transport by H^+^-ATPases and there is a strong positive correlation between H^+^-ATPase activity and salinity stress tolerance in several species (Chen et al., 2007; Bose et al., 2015). Several genes that were down-regulated in the *35S::acdS* line are involved in the maintenance of cellular K^+^, such as Csa02G026480 and Csa08G040240 which encode the high-affinity K^+^ transporter 1, HKT1. Other down-regulated genes included Csa11G065760 (KAT1) that encodes an inward-rectifying K^+^ channel, as well as Csa19G004960, Csa01G002460, and Csa15G002540 which encode the K^+^ transporter, AKT1. AKT1 is involved in the efflux of K^+^ from root stellar and guard cells so that it may be transported into the xylem (Hwang et al., 2013). Increase in cytosolic K^+^ levels, for example via increased H^+^-ATPase activity, triggers programed cell death (Demidchik, 2014). In contrast, Csa11G023970 (potassium transport 2/3) was up-regulated.

In addition to K^+^, changes in the concentration of cytosolic Ca^2+^ with specific amplitude and duration are also vital to plant salinity tolerance. In *A. thaliana* and maize, salt stress leads to a reduction in the concentration of cytosolic Ca^2+^ in roots, while cell cytosolic Ca^2+^ increases in other species (Kader and Lindberg, 2010). One the several genes encoding proteins involved in ion transport that were differentially regulated in roots of the *35S::acdS* line was Csa13G048270, which encodes a component of a cyclic nucleotide gated ion channel (CNGC). Orthologs of this gene encode calcium-permeable, non-selective cation channels that are permeable to Na^+^ and, thus, represent a likely entry point of Na^+^ into the cell (Deinlein et al., 2014). Another gene (Csa20G026050) encoding a Na^+^/Ca^2+^ exchanger similar to CAX7 was also down-regulated. This channel has a high capacity for transporting Ca^2+^ (Carafoli et al., 2001) and promotes leaf senescence in *A. thaliana* through control of ROS homeostasis. Over-expression *CAX7* led to ROS accumulation and accelerated senescence in leaves (Li et al., 2016).

#### Growth and Development

Plants are constantly altering the architecture of their root systems to acclimate to changing soil environments and to optimize resource uptake (Wang et al., 2009). In accordance, a multitude of genes involved is aspects of growth and development were differentially regulated in camelina lines expressing *acdS* or in plants treated with *P. migulae* 8R6.

It has been suggested that roots coordinate growth at the whole plant level during the onset of flowering (Bouché et al., 2016). About 200 genes involved in flower development are expressed in the roots of *A. thaliana* and 595 genes are differentially expressed in roots of plants grown under conditions that induce early flowering (Bouché et al., 2016). In roots of plants treated with *P. migulae* 8R6, a gene encoding Brother of Flowering Locus T and Terminal Flower 1 (BFT) (Csa11g098840) was down-regulated fourfold. BFT proteins repress floral development under salt stress and *BFT* expression is induced by high salinity in an ABA-dependent manner (Ryu et al., 2011).

As with plants treated with *P. migulae* 8R6, roots of the *35S::acdS* line also exhibited down-regulation of genes involved in floral development, such as Csa07G061090 which encodes centroradialis (CEN). *CEN* genes are down-regulated as floral meristem identity genes are up-regulated (Amaya et al., 1999). In addition, 2 camelina homeologs (Csa17G023890 and Csa03G021940) encoding the phosphatidylethanolamine-binding proteins, Mother of FT (MFT) and TFL1, which are positive regulators of seed germination, were also down-regulated. MFT is expressed in response to ABA in the radical-hypocotyl transition zone of the embryo, and *mft* loss-of-function mutants are hypersensitive to ABA during seed germination (Xi et al., 2010).

Csa05G059530 (orthologous to AT1G54560 encoding a myosin family protein with Dil domain 2) and Csa08G054360(annexin 7) were down-regulated. AT1G54560 is involved in pollen growth and annexin 7 is involved in cell organization in response to biotic stress, including salinity stress (Xu et al., 2016). Other genes that were down-regulated in the *35S::acdS* line were Csa10G017960 (SWEET 14) and Csa02G039390 (SWEET13) which encode sucrose efflux transporters. SWEET 14 and SWEET13 are required for proper development of anthers, seeds and seedlings and in modulating the GA response (Kanno et al., 2016). In contrast, some genes that positively regulate growth and development were up-regulated in roots of the *35S::acdS* line, including Csa04G067870 (rubber elongation factor protein; REF). REF is a positive regulator of growth and development in response to water deprivation (Kim et al., 2016).

In the *rolD::acdS* line, genes that influence flower development were also differentially regulated, including Csa01g019170 (JA-responsive 1) which was up-regulated. This protein is involved in the vegetative to reproductive phase transition of meristem responding to various stimuli including JA, cold, salt, and wounding (Xiao et al., 2015). Csa19g023540 (SWEET16) was up-regulated twofold and tight regulation of this gene is important for optimal development under non-favorable and stress conditions in *A. thaliana* (Klemens et al., 2013).

Interestingly, many of the genes that were differentially expressed in the *35S::acdS* and *rolD::acdS* lines or plants treated with *P. migulae* 8R6 were involved in some aspect of inflorescence architecture, flowering or germination; however, their roles in root development have not been studied. It is possible that the product of these genes is transferred to the shoot; however, this needs to be proven. It has also been suggested that the root circadian clock and cytokinin biosynthesis act as a feed-forward loop toward the shoot (Bouché et al., 2016). In *A. thaliana*, FT-like proteins exported from the leaves induce tuberization in potato and bulb formation in onion. These reports indicate that developmental signals originating from the leaf can reach the underground organs and possibly vice-versa (Lee et al., 2013; Navarro et al., 2014; Bouché et al., 2016).

## Conclusion

The mechanism used to deliver acdS affected the degree of protection provided against salt stress. Based on physiological data from this study with NaCl and our previous study with a natural saline condition (Heydarian et al., 2016), endophytic PGPB were more effective than rhizospheric PGPB in maintaining camelina growth under salt stress. In our earlier study, expression of the *acdS* gene in camelina under the control of either the CaMV *35S* and *rolD* promoters enhanced vegetative plant development in the presence of natural saline conditions (Heydarian et al., 2016), as this was also the case in the presence of NaCl (**Figure 1**). However, in both salt stress situations, expression of *acdS* under the direction of the root-specific *rolD* promoter was more effective than the CaMV *35S* promoter. This was also noted in other studies, for example, acdS had a more positive effect on salt and nickel stress tolerance in *Brassica napus* when expressed under the direction of the *rolD* compared to the CaMV *35S* promoter (Stearns et al., 2005; Sergeeva et al., 2006). This was proposed to be due to precise localization of acdS activity and corresponding reduction of ethylene only in the root system where the salt stress is incurred (Stearns et al., 2005).

Gene expression patterns in roots in response to salt stress were also dependent upon the mechanism used to deliver acdS. In roots of both PGPB-treated plants and the *rolD::acdS* line, the number of up-regulated genes was higher than down-regulated genes (1.4-fold in plants treated with *P. migulae* 8R6 and 3.5-fold in the *rolD::acdS* line); while the number of the genes that were down-regulated in the *35S::acdS* line was higher than the number of genes that were up-regulated (1.3-fold). Delivery of acdS via PGPB (*P. migulae* 8R6) mostly affected ethylene and ABA-dependent signaling in positive way, while expression of *acdS* in transgenic lines affected auxin, JA and brassinosteroid signaling and/or biosynthesis more so. Though not a photosynthetic tissue, reduction of ethylene in roots had a positive effect on the expression of genes involved in photosynthesis. The expression of the genes involved in minor carbohydrate metabolism was up-regulated during salt stress, mainly in roots of lines expressing *acdS*. Expression of *acdS* also affected the expression of genes involved in modulating the level of ROS in cells, presumably to prevent cellular damage while permitting ROS-dependent signal transduction pathways to function. Some genome partitioning was observed in camelina as in both *acdS* transgenic lines the number of homeologs of differentially expressed genes assigned to genome III was greater than the other two genomes and in a few cases homeologous genes were regulated oppositely.

## Author Contributions

ZH conducted the research and prepared the manuscript. BG provided the PGPB and *acdS* gene, contributed to experiment design and data analysis. MG and DH are the principal investigators, conceptualized the research, organized funding, supervision of research, and wrote the manuscript.

## Conflict of Interest Statement

The authors declare that the research was conducted in the absence of any commercial or financial relationships that could be construed as a potential conflict of interest.
