# Supplementary figures and images for: Gene Expression Patterns in Roots of Camelina sativa With Enhanced Salinity Tolerance Arising From Inoculation of Soil With Plant Growth Promoting Bacteria Producing 1-Aminocyclopropane-1-Carboxylate Deaminase or Expression the Corresponding acdS Gene
Source: Front Microbiol. 2018 Jun 27;9:1297. doi: 10.3389/fmicb.2018.01297 (PMC6036250; doi:10.3389/fmicb.2018.01297)

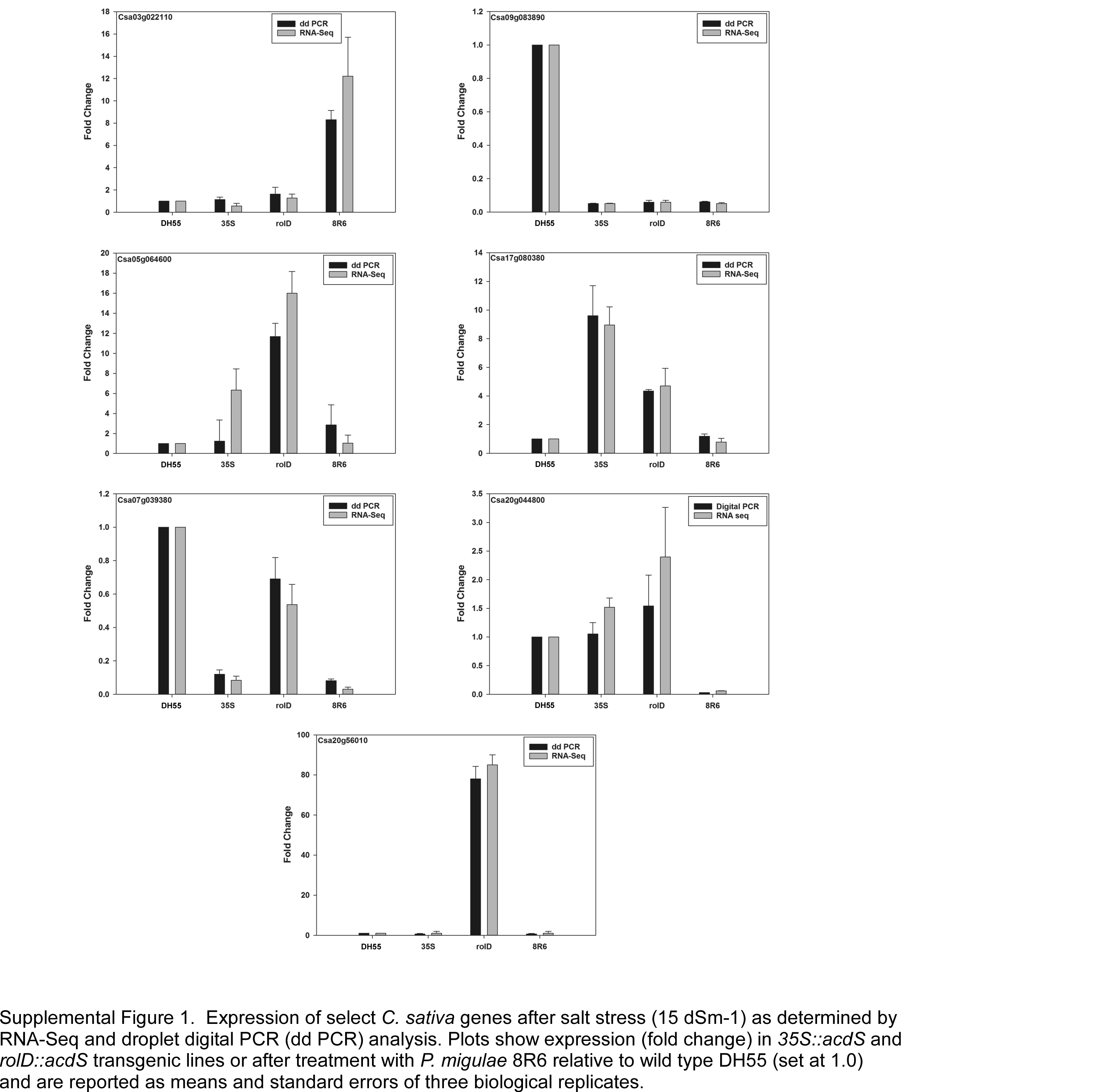

Supplement: Supplementary file 1 [file Image_1.TIF]
