# Supplementary material for: Gene Expression Patterns in Roots of Camelina sativa With Enhanced Salinity Tolerance Arising From Inoculation of Soil With Plant Growth Promoting Bacteria Producing 1-Aminocyclopropane-1-Carboxylate Deaminase or Expression the Corresponding acdS Gene
Source: Front Microbiol. 2018 Jun 27;9:1297. doi: 10.3389/fmicb.2018.01297 (PMC6036250; doi:10.3389/fmicb.2018.01297)
Supplement: Supplementary file 2 [file Presentation_1.pptx]

## Slide 1
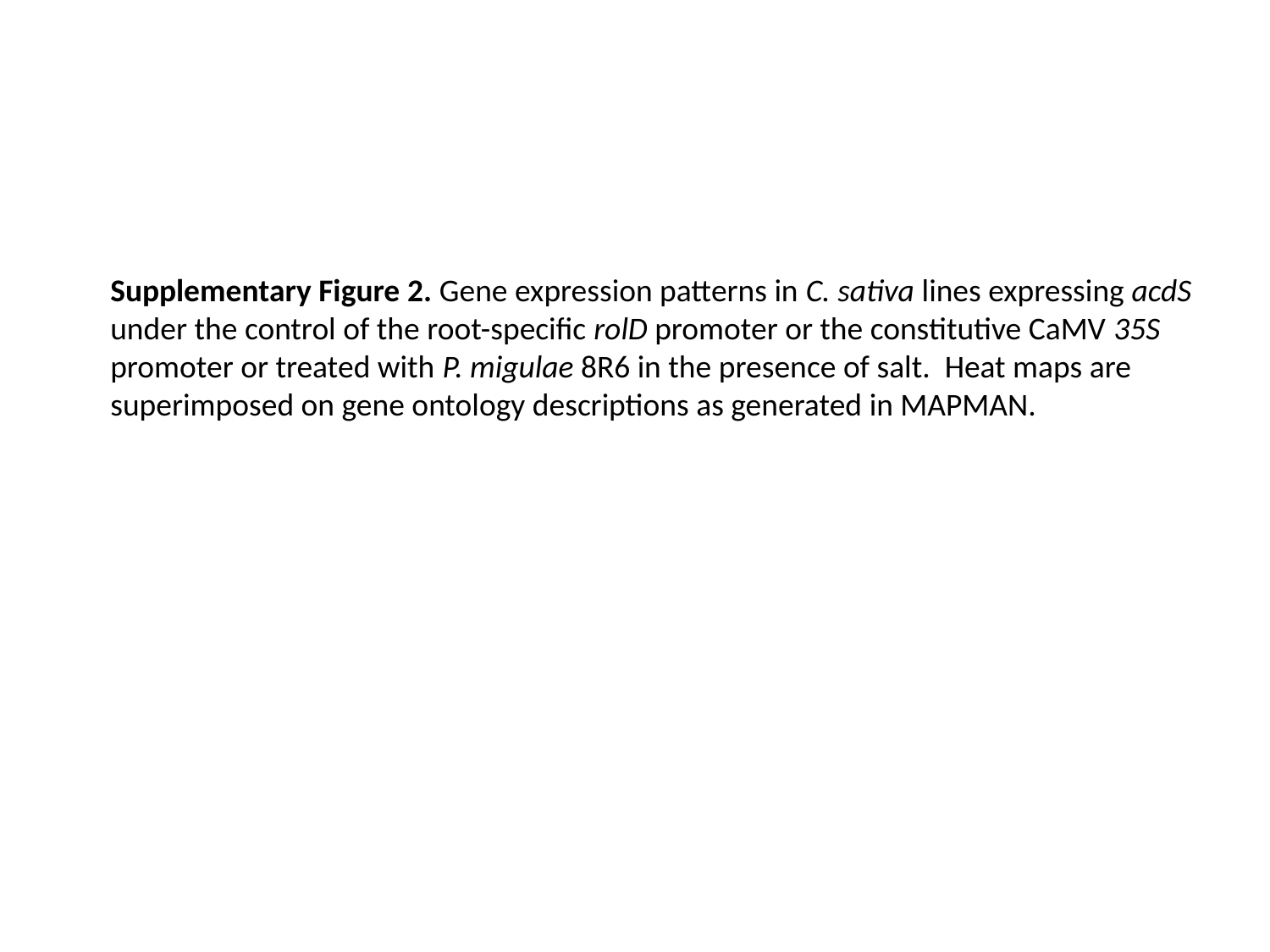

Supplementary Figure 2. Gene expression patterns in C. sativa lines expressing acdS
under the control of the root-specific rolD promoter or the constitutive CaMV 35S
promoter or treated with P. migulae 8R6 in the presence of salt. Heat maps are
superimposed on gene ontology descriptions as generated in MAPMAN.

## Slide 2
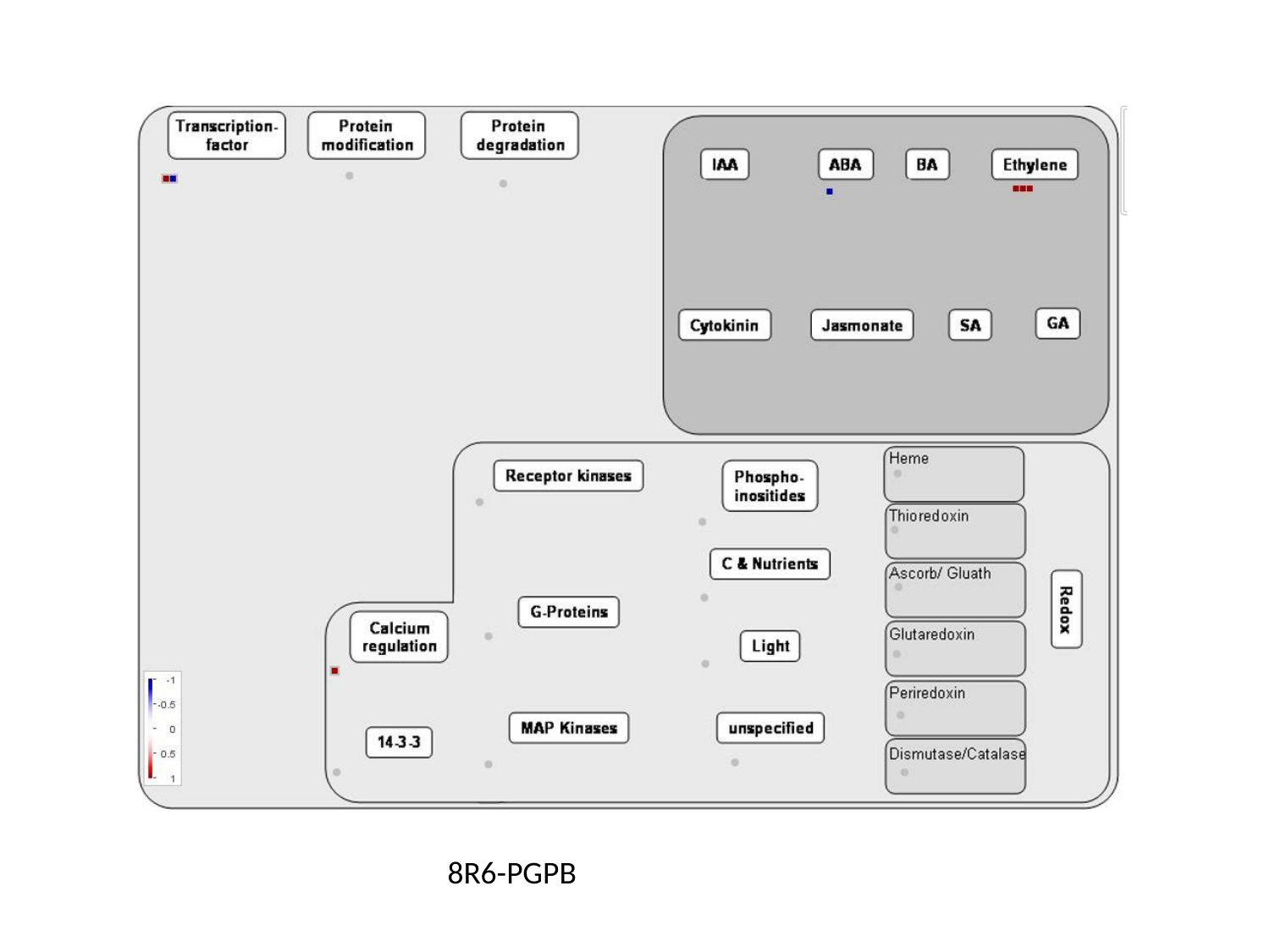

8R6-PGPB

## Slide 3
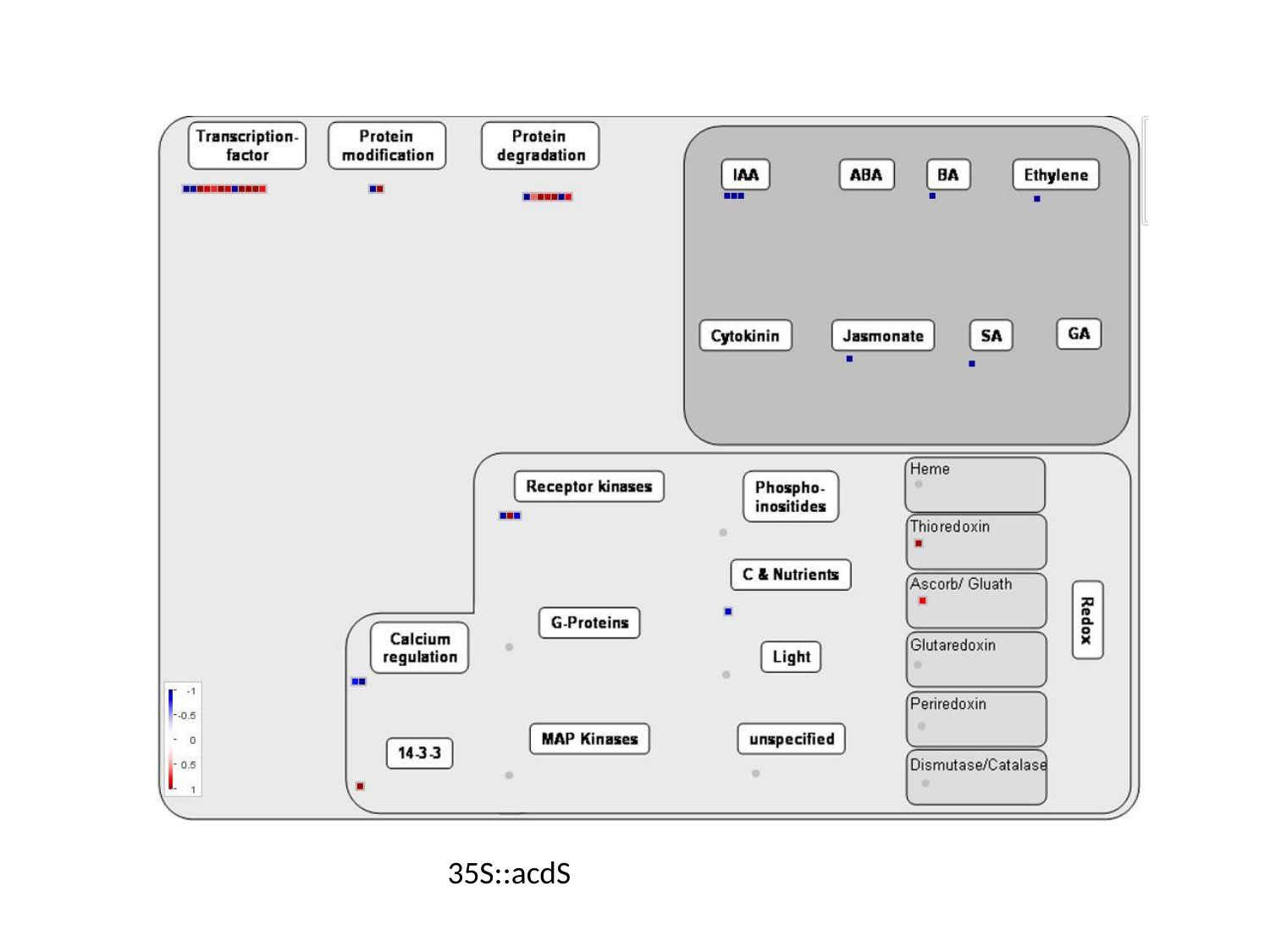

35S::acdS

## Slide 4
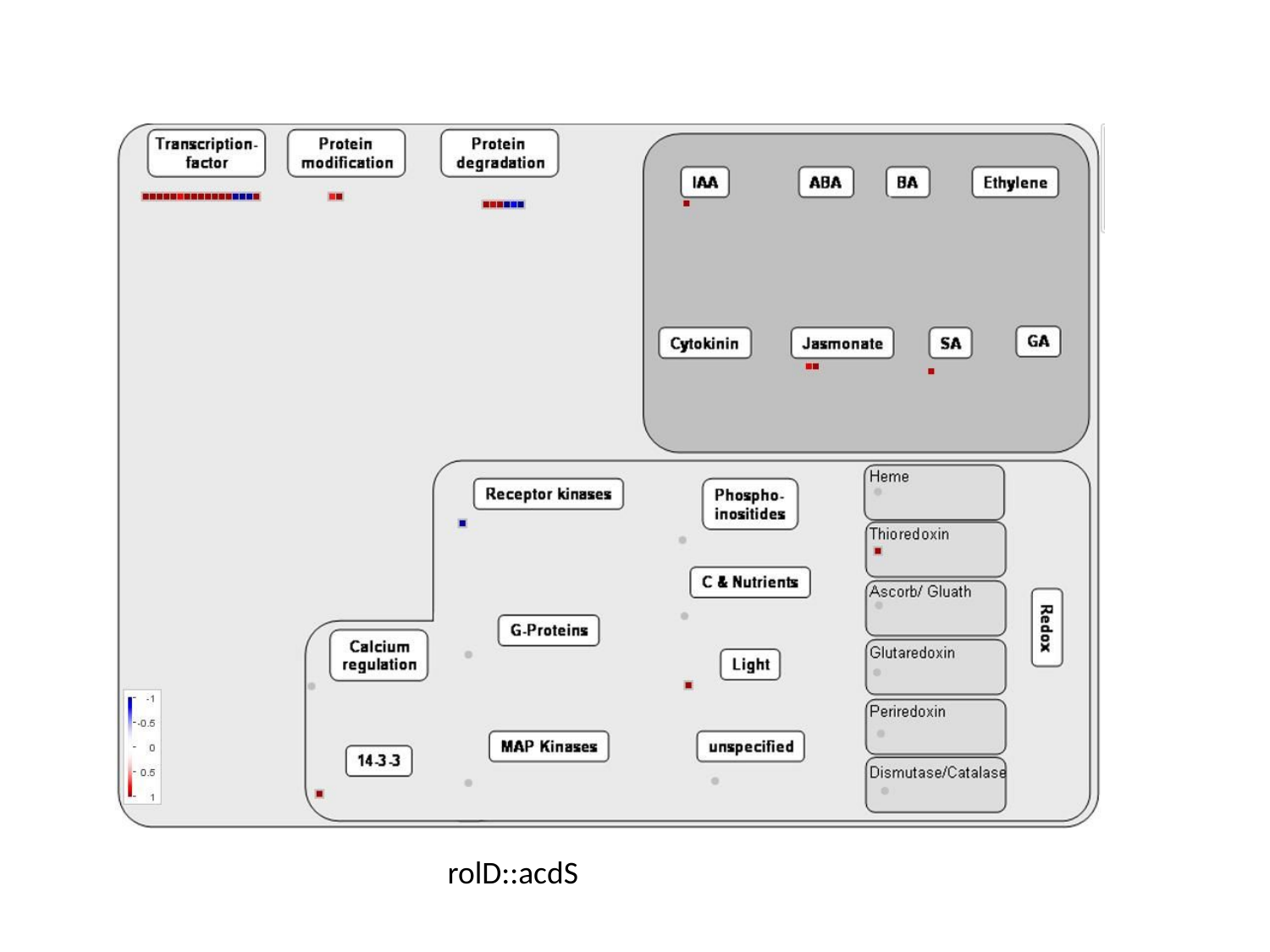

rolD::acdS

## Slide 5
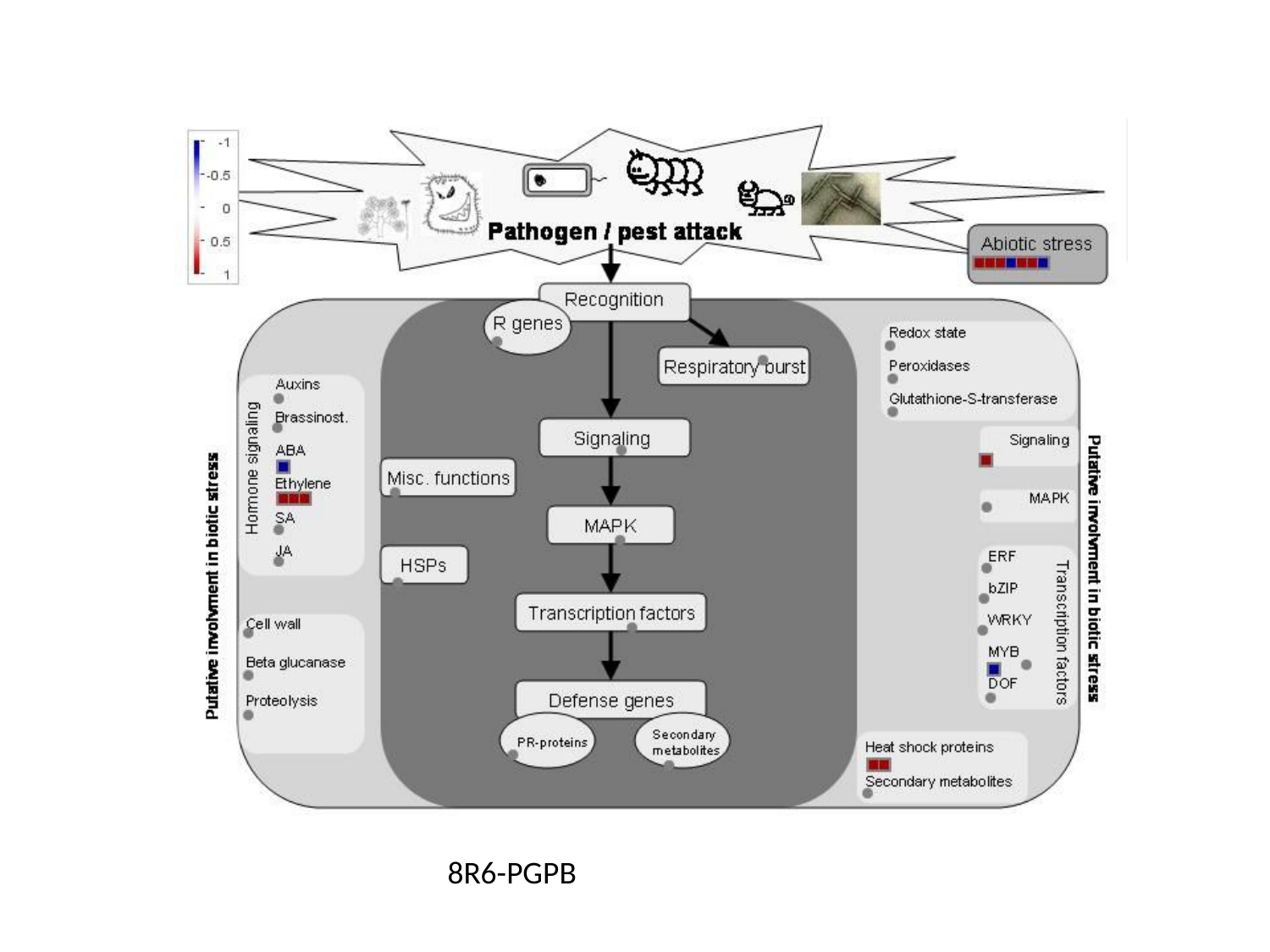

8R6-PGPB

## Slide 6
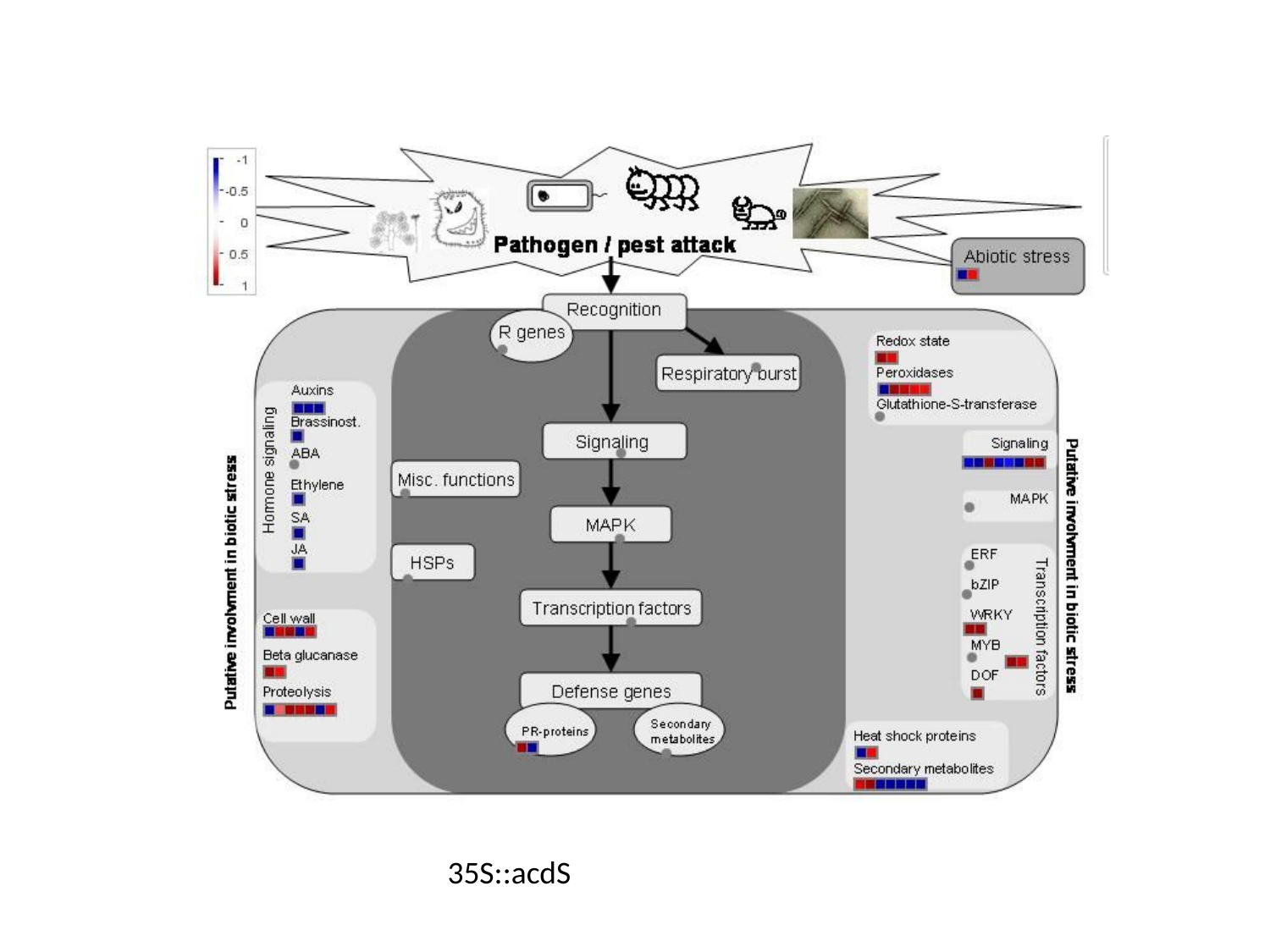

35S::acdS

## Slide 7
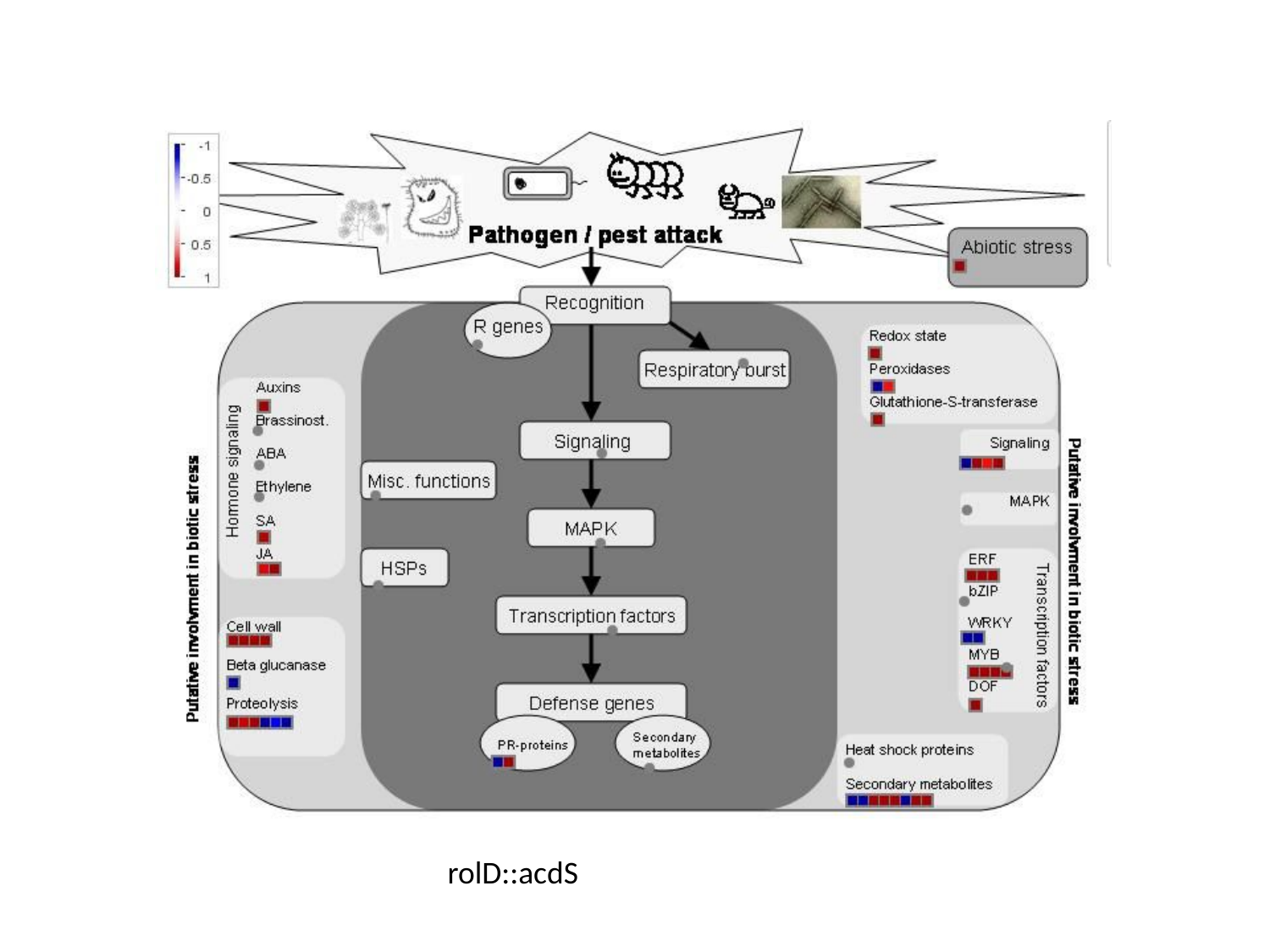

rolD::acdS

## Slide 8
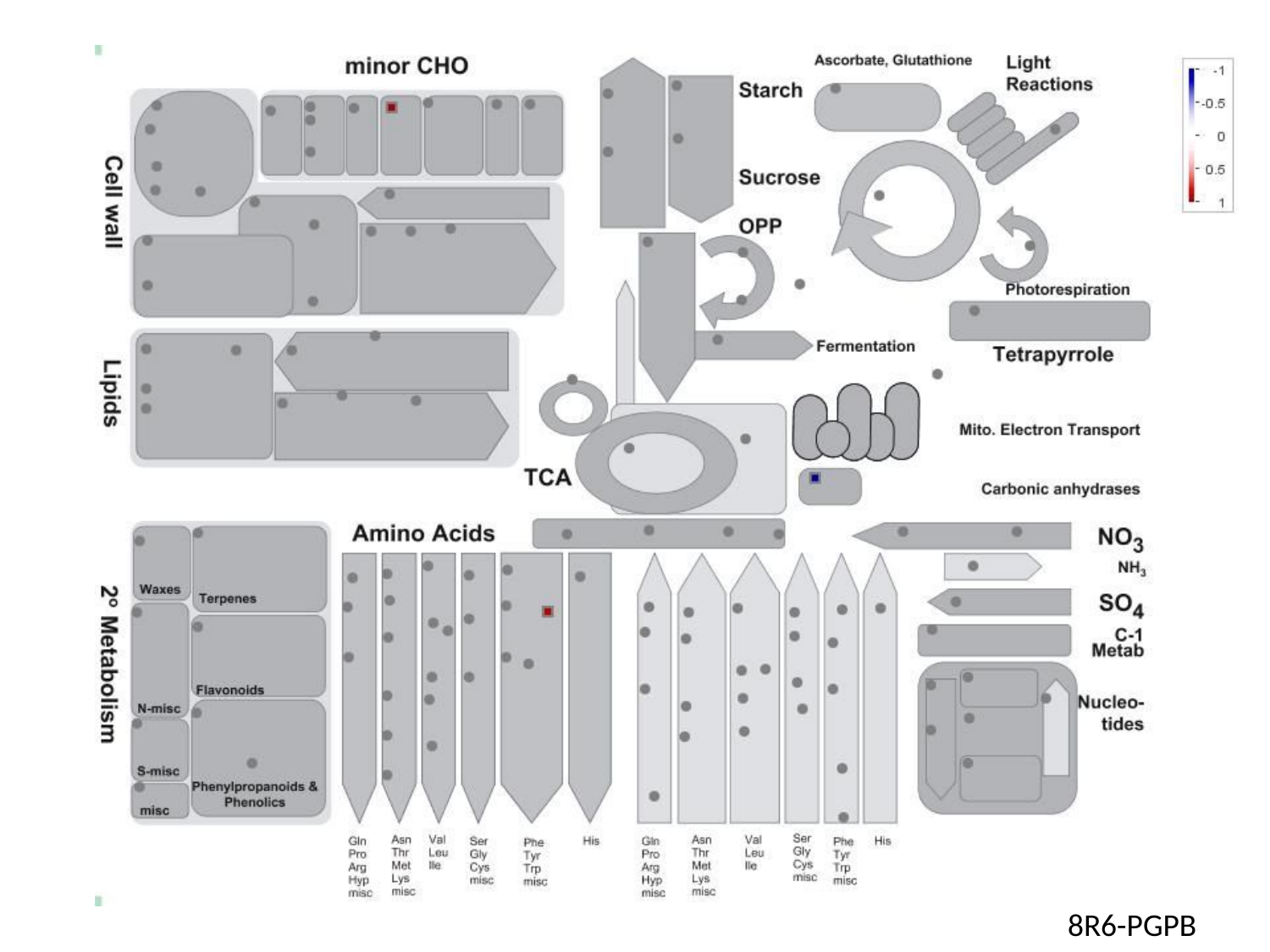

8R6-PGPB

## Slide 9
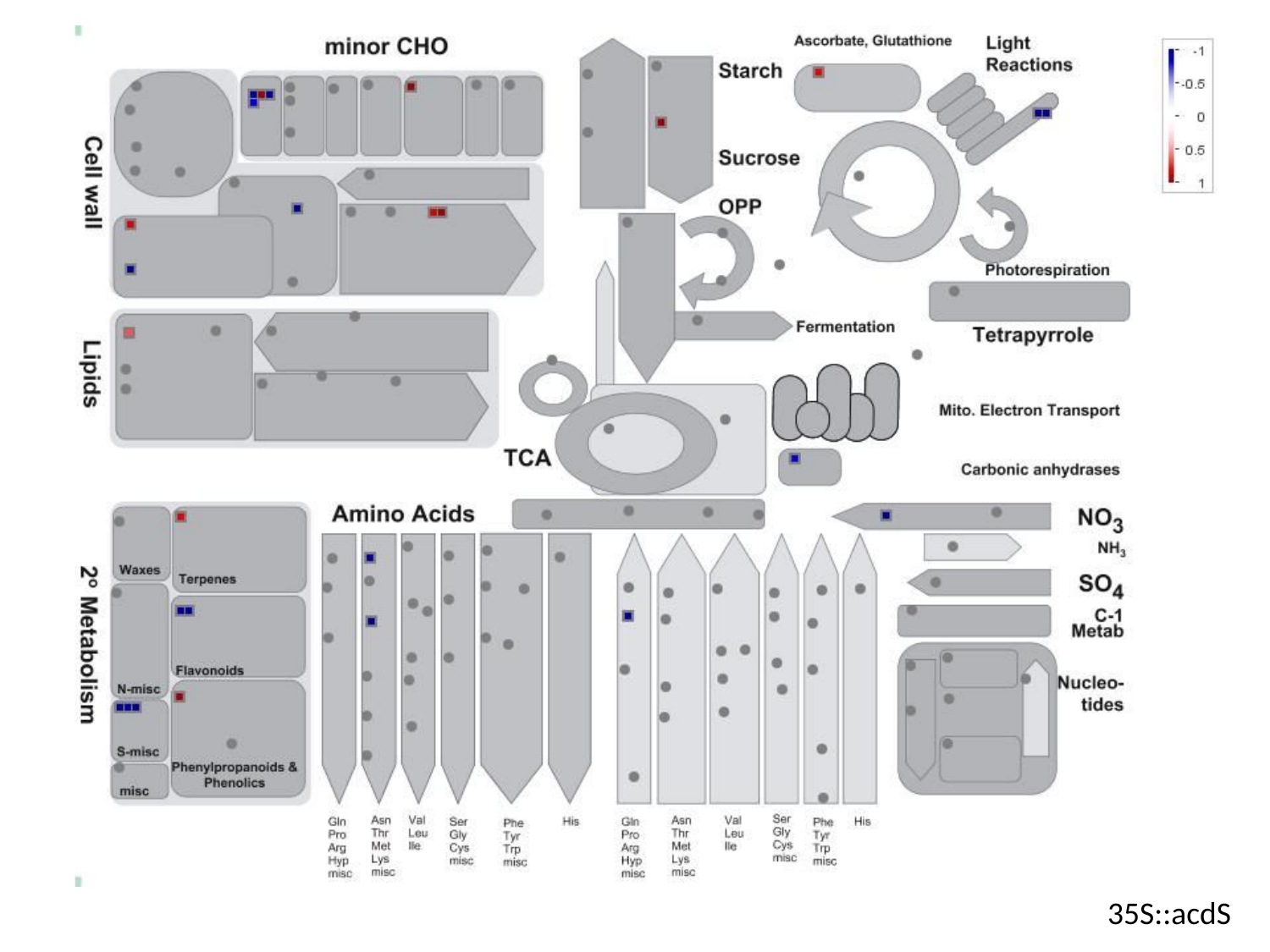

35S::acdS

## Slide 10
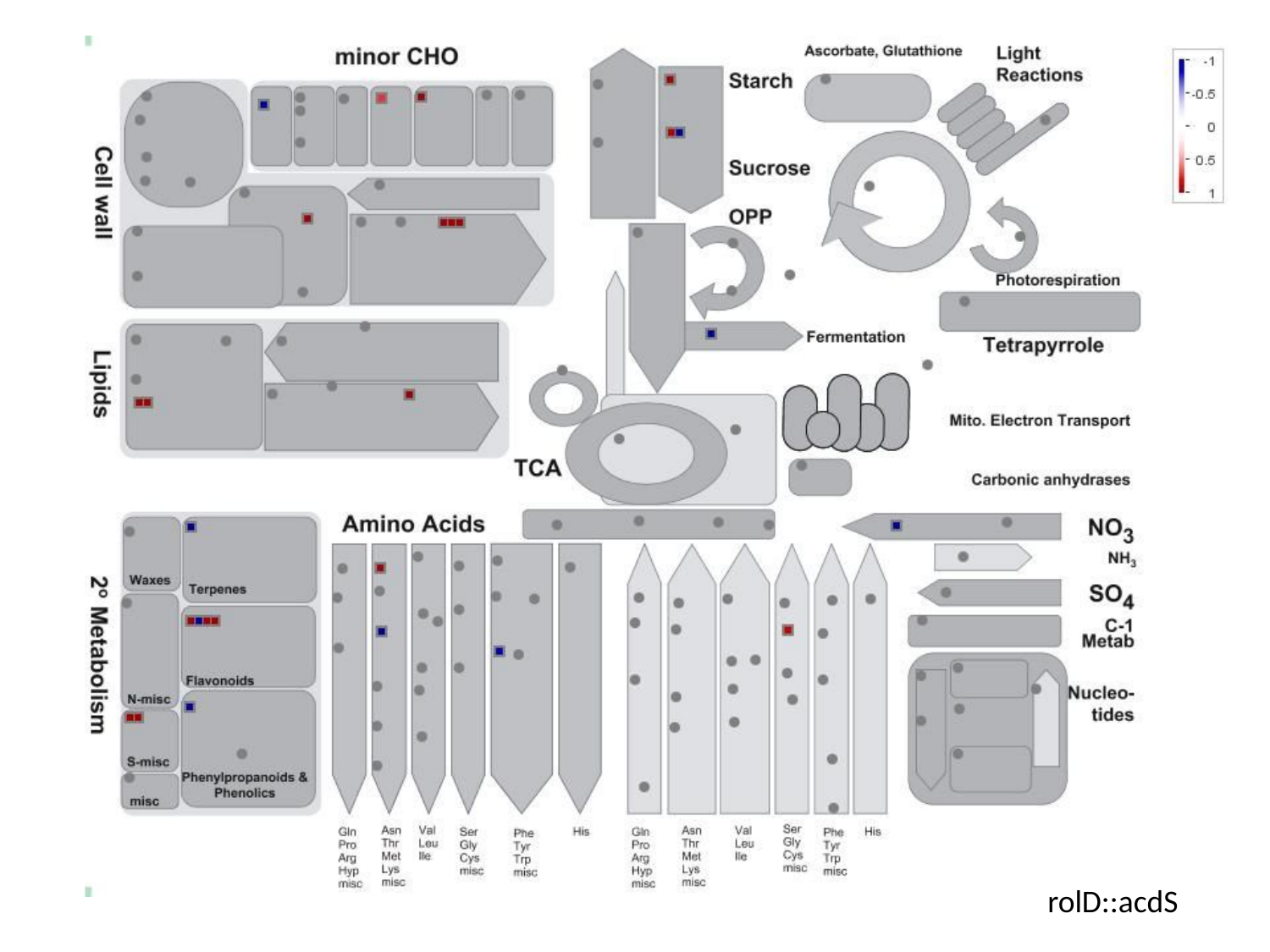

rolD::acdS
